# Huntington’s Disease at the Crossroads of Neurology and Psychiatry: A Case Report

**DOI:** 10.1192/j.eurpsy.2026.11089

**Published:** 2026-07-31

**Authors:** A. Helaoui, S. Khalki, S. Abdelmoumen, R. Hosni, A. Aissa, R. Jomli

**Affiliations:** 1 ”Avicenne’ Psychiatry Department, Razi Hospital, Manouba; 2 Faculty of Medicine of Tunis, Tunis; 3 Ibn Jazzar Faculty of Medicine, Sousse, Tunisia

## Abstract

**Introduction:**

Huntington’s disease (HD) is an autosomal-dominant neurodegenerative disorder combining motor, cognitive, and psychiatric symptoms. Psychiatric manifestations are extremely common, occurring in 73–98% of symptomatic patients, and may precede motor signs by several years. Frequent disturbances include irritability (35–73%), depression (40–50%), apathy (52–76%), while psychosis affects 3–11% of patients.^(1)^

(^1^: Van Duijn et al., 2014).

**Objectives:**

We report a case of acute psychiatric decompensation in a 50-year-old woman with a 10-year history of HD, to highlight how behavioral and psychotic symptoms can exacerbate HD and require multidisciplinary management.

**Methods:**

Case description of a patient seen in the psychiatric emergency department (Razi Hospital), detailing neurologic and psychiatric presentation, management and outcome.

**Results:**

A 50-year-old woman with known Huntington’s disease under olanzapine treatment and a past psychiatric history presented with agitation, irritable mood, and exaggerated affect following a neighborhood conflict.

Examination findings:

- **Psychiatric:** difficult contact; speech stereotypies; persecutory delusion toward neighbor.
- **Neurologic:** choreiform movements, dysarthria, cognitive impairment, motor deficits, confusion.
- **Instinctual functions:** sleep–wake rhythm inversion.

Imaging: CT scan performed and reported as normal.

Management: diazepam 10 mg IM for agitation, followed by maintenance treatment with olanzapine 10 mg/day (HS) and oxazepam 10 mg/day (HS).

6 days later, agitation and psychotic symptoms improved, and she was safely discharged.

**Conclusions:**

This case illustrates that psychiatric crises—with agitation, psychosis, and behavioral disorganization—are frequent in HD, even in patients with prior psychiatric history, and may be precipitated by psychosocial stressors. Recognition of such episodes is essential, as they often stem from fronto-striatal dysfunction and require a tailored multidisciplinary response combining neurology, psychiatry, and caregiver support.^(2)^

(^2^: Reilmann et al., 2014; Martinez-Horta & Kulisevsky, 2019).

**Disclosure of Interest:**

None Declared

